# Trafficking of endoplasmic reticulum-retained recombinant proteins is unpredictable in *Arabidopsis thaliana*

**DOI:** 10.3389/fpls.2014.00473

**Published:** 2014-09-15

**Authors:** Thomas De Meyer, Ann Depicker

**Affiliations:** ^1^Department of Plant Systems Biology, VIB, Plant-made Antibodies and ImmunogensGent, Belgium; ^2^Department of Plant Biotechnology and Bioinformatics, Ghent UniversityGent, Belgium

**Keywords:** molecular farming, antibody production, dense vesicle, ER-derived vesicle, protein storage vacuole, KDEL, seed-specific expression

## Abstract

A wide variety of recombinant proteins has been produced in the dicot model plant, *Arabidopsis thaliana*. Many of these proteins are targeted for secretion by means of an N-terminal endoplasmic reticulum (ER) signal peptide. In addition, they can also be designed for ER retention by adding a C-terminal H/KDEL-tag. Despite extensive knowledge of the protein trafficking pathways, the final protein destination, especially of such H/KDEL-tagged recombinant proteins, is unpredictable. In this respect, glycoproteins are ideal study objects. Microscopy experiments reveal their deposition pattern and characterization of their N-glycans aids in elucidating the trafficking. Here, we combine microscopy and N-glycosylation data generated in *Arabidopsis* leaves and seeds, and highlight the lack of a decent understanding of heterologous protein trafficking.

## Introduction

Recombinant proteins are often produced in eukaryotic host organisms to ensure proper folding, disulfide bridge formation and N-glycan processing. By fusing the protein of interest to an N-terminal endoplasmic reticulum (ER) signal peptide, they co-translationally enter the ER and travel along the secretory pathway, where N-glycosylation takes place on the consensus Asn-X-Ser/Thr motif. The N-glycan composition is of crucial importance for the protein structure, stability, half-life and function, and is primarily determined by the production host (Jacobs and Callewaert, 2009). In molecular farming, where plants are used as production systems, heterologous proteins targeted for secretion typically contain complex-type N-glycans with ß-1,2-xylose and core α-1,3-fucose residues. These are potentially immunogenic and are hence unwanted for therapeutic protein production (Gomord et al., 2005). Therefore, significant efforts went into the development of glyco-engineered production platforms that prevent plant-specific N-glycosylation in the Golgi complex (Gomord et al., 2010; Castilho and Steinkellner, 2012). Alternatively, heterologous glycoproteins can be retained in the ER by adding a C-terminal H/KDEL-tag. Whereas both HDEL- and KDEL-tagged endogenous proteins are found in plants (Napier et al., 1992), the vast majority of heterologously expressed, ER-retained proteins carry the KDEL-tag. Typical for H/KDEL-tagging is the formation of Man_8_GlcNAc_2_ (Man8) N-glycans. However, as ER retention is based on retrograde trafficking from cis-Golgi to ER, the glycoproteins transiently encounter cis-Golgi processing enzymes, such as α-1,2-mannosidase, resulting in partially trimmed N-glycans, such as Man7 and Man6. In addition to the desired N-glycan profile, H/KDEL-tagging can also enhance protein accumulation levels, presumably because the ER is a favorable compartment for protein folding and storage (Fiedler et al., 1997; Petruccelli et al., 2006). However, such an increased accumulation is not always observed (Loos et al., 2011a,b).

In contrast to this black and white distinction between secretion and H/KDEL-mediated ER retention, numerous protein localization studies reported unexpected outcomes. Drawing clear conclusions from these experiments proved hard, because of the different proteins of interest, plant species, tissues, promoters, regulatory sequences, targeting signals and achieved accumulation levels (De Muynck et al., 2010). Moreover, protein trafficking has also been shown to change throughout development (Arcalis et al., 2010; Wang et al., 2012). In this review, we provide a detailed overview of protein localization studies in leaves and seeds of the dicot model plant, *Arabidopsis thaliana*. By limiting ourselves to *Arabidopsis*, we eliminate organismal specificity and highlight tissue (i.e., leaves vs. seeds) and protein specificity in heterologous protein trafficking.

In *Arabidopsis*, most recombinant proteins have been produced in seeds, providing the advantage of long-term storage capacity, high protein content and productivity, and no interference with vegetative plant growth (Stoger et al., 2005; Kermode, 2012). In head-to-head comparisons with the same protein of interest, *Arabidopsis* seeds were more positively evaluated than those of tobacco, petunia and maize in terms of protein accumulation levels (Loos et al., 2011a; Morandini et al., 2011). However, the impact of such comparisons is limited due to the different efficiencies of regulatory sequences and codon usage across organisms. Nevertheless, one of the highest accumulation levels achieved in plants is still that of the seed-produced G4 scFv in *Arabidopsis* (i.e., 36.5% of total soluble protein (TSP) in homozygous seeds) (De Jaeger et al., 2002). De Wilde et al. (2013) showed that VHH-Fc and scFv-Fc accumulation levels of 1% or more in *Arabidopsis* seeds trigger an unfolded protein response, because an enhanced expression of genes involved in protein folding, glycosylation, protein translocation, degradation and vesicle trafficking was observed. However, despite such an altered gene expression profile, *Arabidopsis* seeds often fail to provide a 100% N-glycan site occupancy (Table 1B; 3–18 and 26).

**Table 1:**
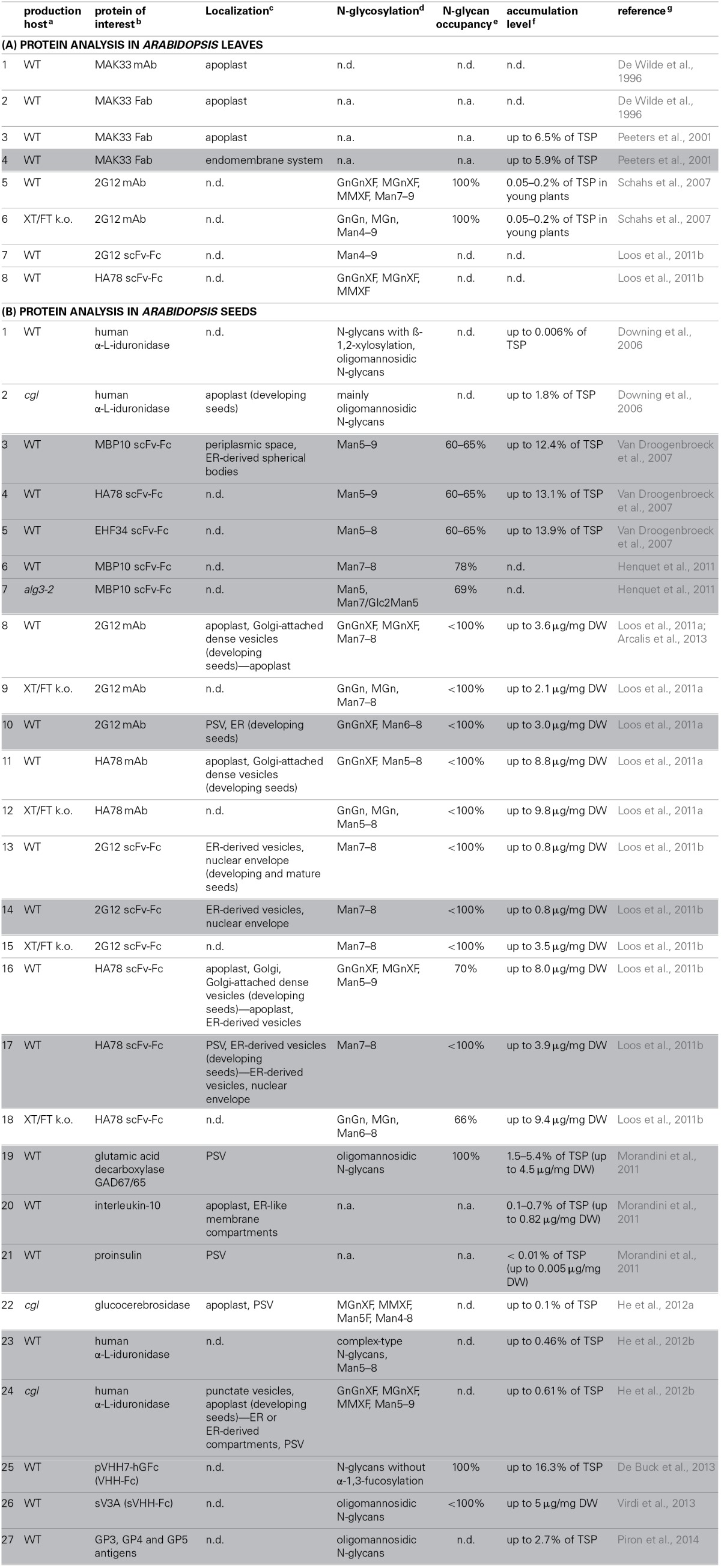
**Overview of recombinant protein production in *Arabidopsis* leaves (A) and seeds (B), in which white boxes indicate recombinant proteins targeted for secretion, and gray boxes correspond to KDEL-tagged proteins**.

*n.d., not determined; n.a., not applicable*.

*^a^alg3-2, α-1,3-mannosyltransferase mutant (Henquet et al., 2008); cgl, complex glycan mutant (Von Schaewen et al., 1993); WT, wild-type; XT/FT k.o., ß-1,2-xylosyltransferase and core α-1,3-fucosyltransferase knockout line (Strasser et al., 2004)*.

*^b^Fab, fragment antigen-binding; mAb, monoclonal antibody; scFv-Fc, single-chain variable fragment fused to a fragment crystallisable; VHH-Fc, variable domain of the heavy chain of the heavy-chain antibody fused to a fragment crystallisable*.

*^c^In mature, dry seeds unless mentioned otherwise*.

*^d^Glc2Man5, Glc_2_Man_5_GlcNAc_2;_ GnGn, GlcNAc_2_Man_3_GlcNAc_2;_GnGnXF, GlcNAc_2_Man_3_GlcNAc_2_XylFuc; Man4–9, Man_4-9_GlcNAc_2_; Man5F, Man_5_GlcNAc_2_Fuc; MMXF, Man_3_GlcNAc_2_XylFuc; MGn, GlcNAc_1_Man_3_GlcNAc_2_; MGnXF, GlcNAc_1_Man_3_GlcNAc_2_XylFuc*.

*^e^Sometimes determined ourselves based on available figures in the corresponding references*.

*^f^In our experience, 1% of TSP corresponds to about 2.5 μg/mg DW. DW, dry weight; TSP, total soluble protein*.

*^g^Studies were ordered chronologically*.

## Trafficking of proteins targeted for secretion

In **leaves**, heterologous proteins that carry an N-terminal ER signal peptide, are efficiently secreted to the apoplast (De Wilde et al., 1996; Peeters et al., 2001) (Table 1A; 1–3) and mainly carry complex-type N-glycans (Schahs et al., 2007) (Table 1A; 5 and 6). Of note, Loos et al. (2011b) found that an anti-hepatitis A virus scFv-Fc (HA78) contained complex-type N-glycans as expected, while an anti-HIV scFv-Fc (2G12) was completely covered with oligomannosidic N-glycans (Table 1A; 7 and 8). Because the authors could not detect antigen-binding activity for this 2G12 scFv-Fc, they postulated that it was not folded properly and activated the ER-associated protein degradation pathway, hence preventing further N-glycan maturation in the Golgi apparatus.

**Figure 1:**
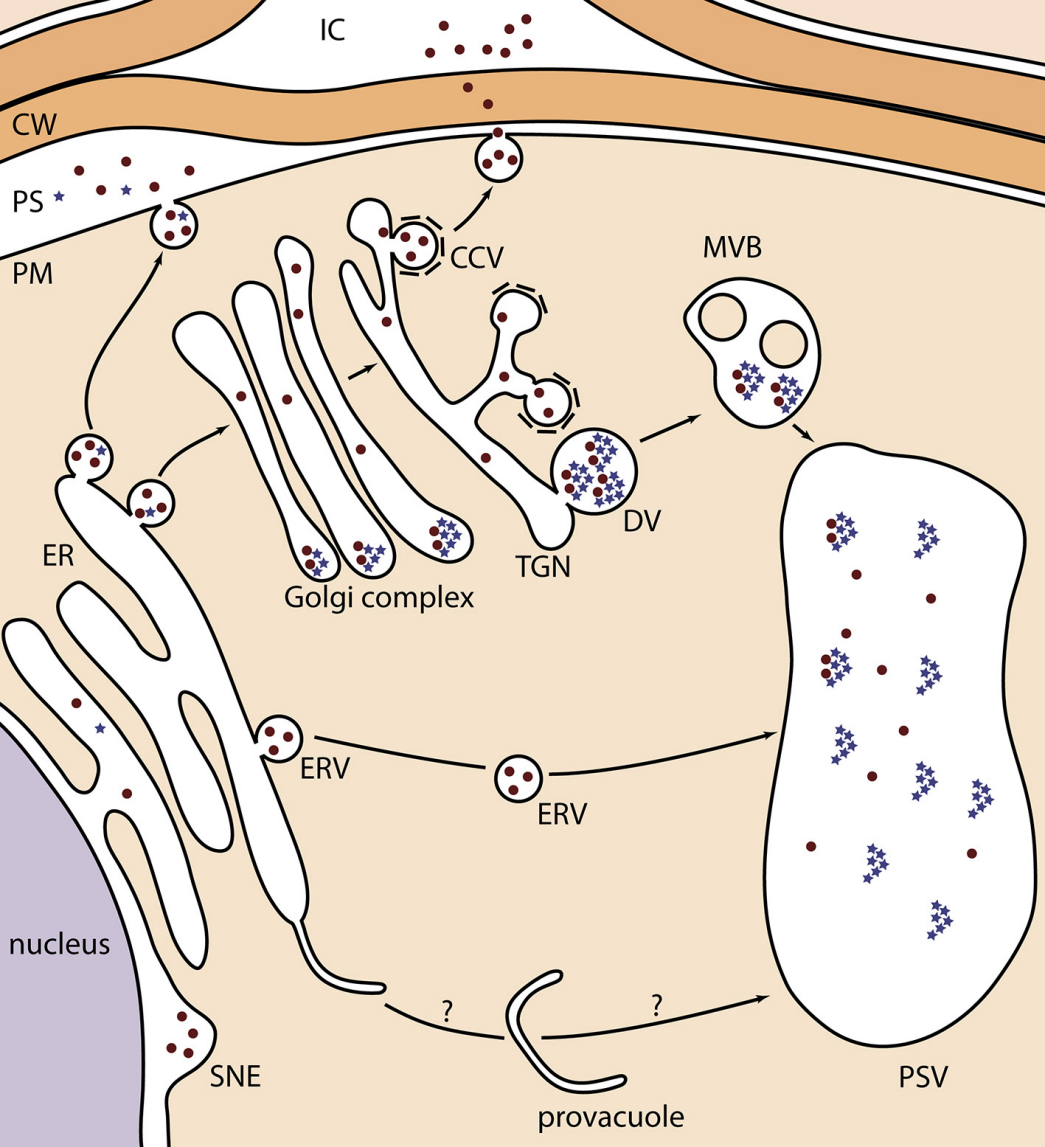
**Schematic representation of the different localizations of KDEL-tagged recombinant proteins in *Arabidopsis* seeds**. Recombinant proteins are depicted as brown dots, and globulin seed storage proteins as blue stars. During their transport, globulins aggregate in the periphery of the Golgi cisternae, from where they bud into DVs toward the PSV. In one occasion, they were observed in the PS because of a disturbed protein trafficking of endogenous proteins (Van Droogenbroeck et al., 2007). The ER-to-provacuole hypothesis, as depicted at the bottom, is based on recent observations of lytic vacuole biogenesis (Viotti et al., 2013). CCV, clathrin-coated vesicle; CW, cell wall; DV, dense vesicle; ER, endoplasmic reticulum; ERV, ER-derived vesicle; IC, intercellular space; MVB, multivesicular body; PS, periplasmic space; PSV, protein storage vacuole; PM, plasma membrane; SNE, swollen nuclear envelope; TGN, trans-Golgi network.

In **seeds**, despite successful examples of protein secretion with complex-type N-glycans, some exceptions stress the lack of a decent understanding of secreted heterologous protein trafficking. For example, HA78 and 2G12 monoclonal antibodies (mAbs) were both found in the apoplast and in electron-opaque Golgi-attached dense vesicles (DVs) in developing seeds (Loos et al., 2011a) (Table 1B; 8 and 11). DVs are distinct from clathrin-coated vesicles that normally mediate protein secretion, and are considered the main pathway for massive seed storage protein transport from the trans-Golgi network to the protein storage vacuole (PSV) (Robinson et al., 2005; Vitale and Hinz, 2005; Otegui et al., 2006; Wang et al., 2012) (Figure 1; blue stars). Their electron-opaque content reflects the aggregated state of the storage proteins. Possibly, the highly abundant storage proteins, such as globulins, exhibit a dominant sorting effect that leads to partial trapping of the heterologous proteins in DVs. A similar mechanism, imposed by endogenous seed storage proteins, has been proposed for recombinant phytase in ER-derived prolamin bodies of rice endosperm (Drakakaki et al., 2006). In agreement with the co-sorting hypothesis to the PSV via DVs in *Arabidopsis*, glucocerebrosidase that was targeted for secretion, was mainly located in the apoplast and to a minor extent in PSVs in mature seeds (He et al., 2012a) (Table 1B; 22). Alternatively, partial mislocalization of secreted proteins to DVs and PSVs can also be explained by the presence of cryptic vacuolar specific sequences (Jolliffe et al., 2005).

Both the HA78 and 2G12 mAbs were produced as scFv-Fc moieties, using the same targeting and regulatory sequences (Loos et al., 2011b) (Table 1B; 13 and 16). On the one hand, labeling in apoplast and Golgi-attached DVs was obtained for HA78 scFv-Fc (identical as for HA78 mAb) in developing *Arabidopsis* seeds. In mature seeds, PSVs were devoid of label, so the question remains where the DV-localized HA78 scFv-Fcs of the developing embryos ended up. Instead, the final destinations of HA78 scFv-Fc were the apoplast and “globular, membrane-delimited structures of around 200 to 400 nm in diameter.” The latter were termed ER-derived vesicles (ERVs), because ribosomes were observed on their surface, but their specific formation in later developmental stages was unclear. This dual deposition pattern was in accordance with the presence of both complex-type and oligomannosidic N-glycans. On the other hand, 2G12 scFv-Fc exclusively contained Man7 and Man8 N-glycans, and was observed in ERVs and the swollen nuclear envelope. This aberrant localization is in agreement with the proposed improper folding of 2G12 scFv-Fc (see above in *Arabidopsis* leaves).

## Trafficking of proteins targeted for ER-retention

Only one study has been performed in *Arabidopsis* **leaves**, in which a KDEL-tagged Fab fragment was detected intracellularly, most likely in the endomembrane system (Peeters et al., 2001) (Table 1A; 4).

In **seeds**, only Loos et al. (2011a) conclusively demonstrated successful ER retention, more in particular for a minor fraction of KDEL-tagged 2G12 mAb (Table 1B; 10). All other studies describe distinct deposition patterns (Figure 1). First, the most prevalent observation is the formation of ERVs, in a process that is not fully understood (Van Droogenbroeck et al., 2007; Loos et al., 2011b; Morandini et al., 2011; He et al., 2012b) (Table 1B; 3, 14, 17, 20 and 24). Their origin resembles KDEL-vesicles (KV) of *Vigna mungo* seeds, by which SH-EP, a KDEL-tagged vacuolar proteinase, is shuttled from the ER to the PSV upon germination (Toyooka et al., 2000). Moreover, the C-terminal KDEL-tag of SH-EP was shown to be essential for KV formation (Okamoto et al., 2003). Similarly, after producing GFP-KDEL in tobacco leaves, so-called protein bodies were observed in most transformants with a GFP accumulation level of at least 0.2% of TSP (Conley et al., 2009; Gutiérrez et al., 2013). Taken together, it seems that the KDEL-tag ensures a local protein build-up in the ER lumen, from which ERVs, KVs or protein bodies are formed. From results obtained in mature *Arabidopsis* seeds, two hypotheses were made. On the one hand, ERVs can represent the end-stage of heterologous protein trafficking (Van Droogenbroeck et al., 2007; Morandini et al., 2011; Loos et al., 2011b), which sometimes are observed together with equal amounts of protein deposited in the swollen nuclear envelope (Loos et al., 2011b) (Table 1B, 14 and 17). On the other hand, ERVs can mediate a Golgi-independent pathway to the PSV. This was demonstrated by the EndoH sensitivity of the GAD67/65 glycoprotein (Morandini et al., 2011) (Table 1B; 19) and the large fraction of oligomannosidic N-glycans on 2G12 mAb (Loos et al., 2011a) (Table 1B; 10). A similar ER-derived Golgi-independent pathway toward the PSV has been described for endogenous seed storage proteins in pumpkin seeds, where the shuttle vesicles were termed precursor-accumulating (PAC) vesicles (Hara-Nishimura et al., 1998). The authors hypothesized that the PAC pathway has evolved for efficient, massive transport of unglycosylated seed storage proteins to the PSV. Of note, vacuole biogenesis might also represent an ER-to-vacuole route, because the ER was recently proposed as the main membrane source for lytic vacuole formation (Viotti et al., 2013). Although such a mechanism has not yet been observed for PSVs, it is tempting to state that some ER-retained heterologous proteins are trapped into a PSV precursor in a similar process (Figure 1).

Second, partial leakage to the Golgi has been observed because KDEL-tagged proteins accumulated in the apoplast (Morandini et al., 2011) (Table 1B; 20) or carried complex-type N-glycans (Loos et al., 2011a) (Table 1B; 10). Such ER leakage has also been described in *Medicago* and tobacco (Triguero et al., 2005; Petruccelli et al., 2006; Abranches et al., 2008). The authors suggested several factors that might influence successful ER retention. For example, one should consider the amount of KDEL-tags per assembled molecule, and the accessibility and integrity of the KDEL-tag. Remarkably, based on western blot analysis of subcellular fractions, He et al. (2012b) suggested a Golgi-dependent route toward the PSV for KDEL-tagged human α-L-iduronidase (Table 1B; 24). Unfortunately, electron microscopy localization studies on mature seeds confirming this hypothesis, were lacking.

Third, after producing KDEL-tagged MBP10 scFv-Fc in *Arabidopsis* seeds, an electron-opaque periplasmic space (PS) between the plasma membrane and cell wall was observed in which most of the MBP10 was deposited (Van Droogenbroeck et al., 2007) (Table 1B; 3). Moreover, MBP10 exclusively contained oligomannosidic N-glycans, pointing to a Golgi-independent route from the ER to the PS. Because ER-resident proteins, such as calreticulin and binding protein, were also present in the PS, and because of the very high MBP10 accumulation level (up to 12.4% of TSP), the authors proposed an overcharge of the ER storage capacity. Interestingly, globulin storage proteins were also deposited in the PS.

In several other studies, localization experiments were out-of-scope. However, N-glycan analyses were performed and only oligomannosidic N-glycans were detected (Henquet et al., 2011; De Buck et al., 2013; Virdi et al., 2013; Piron et al., 2014) (Table 1B; 6, 7 and 25–27). Although the authors probably assumed successful ER retention, we conclude that, based on the aforementioned detailed localization studies, these KDEL-tagged glycoproteins can also reside in ERVs, the PSV (by bypassing the Golgi complex) or the PS.

## Concluding remarks

It is critical for recombinant protein production that the platform is reliable and predictable in terms of product quality. In this respect, the ER retention of KDEL-tagged recombinant proteins in *Arabidopsis*, has proven to be unpredictable, and similar observations were made in other plant production systems. Therefore, H/KDEL-tagged proteins, produced in plants, should always be analyzed in terms of final destination and N-glycan composition, unless of course, if the actual N-glycan composition is of less importance (e.g., for diagnostic proteins) and does not influence the final product performance.

Obviously, additional investigations are needed. For example, one could analyse the influence of the protein accumulation level by comparing protein localizations in low and high expressing lines. Alternatively, the promoter sequences used might also severely impact the observed protein localization. To this end, promoters with different temporal and spatial expression patterns, especially during seed development, could be worthwhile to study. Further, deletion and swapping experiments can reveal whether particular protein domains of the heterologous protein contain certain localization motifs resulting in the lack of ER retention. Finally, most of the manuscripts discussed here, did not verify KDEL-tag accessibility or integrity. For future reports on H/KDEL-tagged protein production in plants, it would thus be valuable to include, for example, western blot or immunoprecipitation analyses with commercially available anti-H/KDEL antibodies. In case recognition by these antibodies fails, linker sequences can be used to improve H/KDEL-tag accessibility. We conclude that such investigations will result in a much better predictability of the ER retention of overexpressed H/KDEL-tagged proteins in plants, and eventually contribute to the further establishment of the field of plant molecular farming.

### Conflict of interest statement

The authors declare that the research was conducted in the absence of any commercial or financial relationships that could be construed as a potential conflict of interest.
